# Cases of Diseased Vertebræ

**Published:** 1816-10

**Authors:** R. G. A. Collingwood

**Affiliations:** Physician to the Universal Medical Institution, &c. St. George's in the East, London.


*For the London Medical and Physical Journal.*

*Cases of Diseased Vertebræ;* by R. G. A. COLLINGWOOD, M.D. F.R.P.S. Ed. Physician to the Universal Medical Institution, &c. St. George's in the East, London.

ON the 10th of April, 1812, I was consulted in the case of Wm. G., aged 14 years. He complained of severe pain in his "back and belly," and also in some of the larger joints. The state of the pulse was such as might indicate venæsection; but, as he was under the necessity of going to sea next morning, it was not then had recourse to; and, conceiving that the disease might be of a rheumatic nature, some of the usual medicines were ordered him, to take during the voyage. On his return from sea, in the month of May, he still complained of the pains in his back and bowels, accompanied with severe head-ach. The pulse continuing full and strong, ℥xij. of blood were taken from his arm, which, upon cooling, exhibited a thick inflammatory crust. Purgatives, diaphoretics, &c. were employed successively without any advantage. The patient, on being questioned respecting the causes which might have produced his complaints, said that he had not been much exposed to cold or wet, but, having displeased the master of the vessel to which he belonged, the master struck him violently with his foot on the lower part of the back, which disabled him from walking for a short time. About the 2d of June, he began to complain of a numbness of his legs; and the pain in his back and bowels became so severe as to prevent him from resting either night or day. Leeches were applied to that part of the back where the pain was most acute, and afterwards repeated blisters, without any apparent advantage. From my first being called in, I had frequently examined the course of the spine, but could find nothing different from the natural appearance, until about the 2d of July, when I thought I could perceive a slight rising or protuberance over the eighth and ninth dorsal vertebræ, inclining towards the inferior angle of the scapula of the right shoulder. This was, however, so slight, that other professional men, who made examination at the same time, were of opinion that it was only imaginary. I had stated my my opinion, that there existed a diseased state of the vertebræ; and in this I was now fully confirmed by the patient complaining of increased pain, whenever pressure was made upon the prominent part.

The concomitant symptoms were, a painful sensation extending from the diseased vertebræ round the body, but most distressing about the scrobiculus cordis; inability to pass the fœces, without the frequent use of clysters and purgatives; dryness of the skin, below the seat of the disease; and, although sudorifics brought out a copious sweat on the superior parts of the body, the inferior were never found to perspire.

Before the end of the month, the lower extremities were wholly deprived of the power of motion, and nearly of sensation. The patient lay almost constantly on his back in bed; but, whenever obliged to sit up for a few minutes, his toes could only be brought to rest upon the ground, the ankles being in a state of rigidity. I now recommended a seton to be passed on each side of the diseased vertebræ, but this was not consented to till the expiration of more than a week, when they were introduced, and a copious discharge obtained for upwards of two months, yet the patient was no better, and would have gladly laid them aside. I, however, persuaded him to allow them to remain a month longer, about which time he began to feel a gradual abatement of the pain in his abdomen and pit of the stomach. His legs, whilst in bed, would be seized with shooting pains and involuntary startings; or, if sitting, would be suddenly brought across each other, and drawn up under the seat, and, when attempting to walk, the legs were moved in a different direction to that intended. The rigidity of the ankles now gradually diminished, and he every day appeared to be getting better. A distressing head-ach, however, at this time began to trouble him much, yet his convalescence daily increased, and he could, by the end of October, walk across his room, by the help of crutches; and his legs recovering the power of sensation also, he experienced a pleasing warmth in them, which was followed by a gentle moisture on the surface. The acute pain in his head now entirely left him, but was followed by a heavy dull sensation, strabismus, and dilatation of the pupils of the eyes. The pulse, at the beginning of the disease, was full and strong, as it advanced weak and variable, and now became quick and small. These symptoms might seem to indicate some affection of the brain, and a practitioner called in my absence from home, who pronounced it hydrocephalus, to which it had some resemblance. Blisters to the head, head, and a course of hydrargyri submurias, were employed for a fortnight. During this time, his other symptoms still kept gradually wearing off, and he was soon able to go into the open air, and use a little exercise. The affection of the head also by degrees disappeared. He was able to go to sea by the 1st of March, 1813; and is at this time a stout and healthy young man.

John Thorl, a German sailor, ætatis 35, complained of great weakness of his back and lower extremities, pain round the trunk of the body, and a sense of straitness about the scrobiculus cordis. In walking, his legs seemed rather to be drawn after him, than lifted forward in the usual manner; and, when sitting, the toes turned inwards, and rested upon the ground with the heels raised. On examining the course of the spine, the eleventh dorsal vertebra was found to be considerably higher, or more prominent than the rest, and appeared as if dislocated, the spinous process almost cutting through the skin. From the accounts of this patient, the disease had been present about eight months, but he could assign no cause for its production, said that he had never received any sprain or fall on that part of the body, and always previously enjoyed a good state of health. Prior to his consulting me, he had been in two hospitals without experiencing any benefit from different modes of treatment. I ordered a seton to be passed on each side of the diseased bone; and these were moved twice a day, and occasionally dressed with the ceratum sabinæ, so as to keep up a copious discharge from the parts. Internally he took the powder of cinchona, a mild mercurial alterative, and a few palliative medicines. For the first month, little or no benefit was experienced from the above mode of treatment; but, before the end of the second month, he found himself considerably stronger, was able to walk a little every day, his appetite improved, and he began to gain flesh. After six weeks longer perseverance in the same means, he found himself quite recovered. One seton was now removed, and the other allowed to remain a few days longer, and then also taken away. Purgatives were occasionally exhibited, a nutritive diet recommended, and he now finds his complaints completely removed, his general health being as good as at any former period of his life. From the extreme diseased state of the vertebra, and very fœtid smell of the matter discharged, frequently containing small spiculæ of bone, it is probable that exfoliation took place. In the approach to convalescence, he wriggled much in walking, and, in lifting his feet, had no seeming voluntary power to replace them again on the ground, but had the paralytic shake and want of government so accurately described by the celebrated Pott.

In the case of Mary Dinning, a child, five years old, there were some symptoms of scrofula, and her mother had also some of the appearances of the scrofulous habit. The principal complaints under which this patient laboured were deformity or curvature of the superior vertebræ dorsi, weakness of the back and loins, inability to walk firmly, and a retrocedent posture of the head, as if too heavy for the neck. Setons were recommended, but the parents of the child would not allow of them. Stimulating embrocations were then ordered to be applied over the whole course of the spine by long-continued frictions, a small pad over the distortion of the spine, and a bandage round the body; and rest in the horizontal posture strictly enjoined. The pulv. cinchonæ and mild mercurial preparations were given, under which means, after three months' perseverance, she completely recovered, and is now straight, active, and healthy.

John D., ætatis 32, was, about three years ago, affected with many symptoms supposed to resemble paraplegia, and at length entirely lost the use of his lower extremities. About twelve months ago, I was called to see him, and found him sitting with his heels drawn up under the chair, his toes resting on the ground, and pointing inwards, with rigidity of the ankles. He did not complain of pain in any particular part of the body, except a slight degree in the back, and an oppression about the scrobiculus cordis. The power of sensation in the limbs was yet tolerably perfect; and they retained their usual bulk, Previous to my visiting him, he had had recourse to almost the whole of the medical and surgical advice of the town in which he resided. Purgatives, blisters, external and internal stimulants, large issues over the exit of the great sciatic nerves, had been repeatedly employed, without the least benefit. I requested to see his back, when I discovered a small protuberance over the ninth dorsal vertebra, in which, upon pressure, he experienced a slight degree of pain. I recommended setons to be passed near what I considered the seat of the disease; but he would not submit to their use, as he said he had experienced so much pain from the issues before employed, without any advantage. After lingering in this state about nine months after I first saw him, and using every remedy excepting setons, I am informed that he died.

In four cases of diseased spine (which have lately come under my cognizance) in children of two, three, five, and eleven years of age, where the horizontal posture, with rest, as lately recommended, were employed for a considerable length length of time, three of the children died, the eldest with many of the symptoms of phthisis pulmonalis. The youngest child is still alive, but not in the least recovered.

Some well-marked cases, successfully treated, with the observations of recent authors that I have not yet seen, and the advancement in medical knowledge, may enable me to offer a few observations on the subject at a future period.

*June* 19, 1816.

